# Common Iliac Artery Mycotic Pseudoaneurysm Associated with a Prevertebral Infection: A Case Report

**DOI:** 10.5811/cpcem.1348

**Published:** 2023-10-18

**Authors:** Will Davis, Christopher Greene, Brendan Anzalone

**Affiliations:** University of Alabama at Birmingham, Department of Emergency Medicine, Birmingham, Alabama

**Keywords:** *case report*, *mycotic pseudoaneurysm*, *Proteus mirabilis*

## Abstract

**Introduction:**

Mycotic pseudoaneurysms are rare but severe sequelae of an arterial wall infection. If undiagnosed and untreated they can lead to significant morbidity and mortality through complications such as arterial rupture or dissection.

**Case report:**

This report details the case of a 64-year-old-male who developed a left common iliac artery mycotic pseudoaneurysm from *Proteus mirabilis*, which was associated with a prevertebral abscess. The patient presented with isolated, left lower extremity edema and intermittent fevers. The case is unique in both the pathogen (*P mirabilis*) and in its association with presumed direct arterial wall infection from an adjacent prevertebral abscess.

**Conclusion:**

The obscure presentation highlights the need for a high clinical suspicion of such a diagnosis when a patient presents with a certain constellation of symptoms and the right predisposing risk factors in their history.

CPC-EM Capsule What do we already know about this clinical entity?

*Mycotic pseudoaneurysms (MPA) are relatively rare and difficult-to-diagnose clinical entities that can lead to severe morbidity and mortality.*

What makes this presentation of disease reportable?

*The presentation described in this case highlights a unique presentation of a MPA associated with a prevertebral infection*.

What is the major learning point?

*This case demonstrates the limitations of some initial diagnostic tests and the need to consider further testing if clinical suspicion remains for a diagnosis.*

How might this improve emergency medicine practice?

*Clinical practice can be improved through this case review by reminding clinicians to consider the benefits and limitations of common diagnostic testing.*

## INTRODUCTION

Bacterial and fungal infections of an arterial wall can lead to significant morbidity and mortality through the creation of aneurysms and pseudoaneurysms and the complications they entail.^1^ The term “mycotic” is used to encompass both fungal and bacterial causes of infection.^2^ These infections can occur in any artery, and case reports describe several locations.^3^ ^–^ ^5^ These aneurysms and pseudoaneurysms are commonly attributed to septic emboli from infective endocarditis or direct injury (iatrogenic and traumatic) to the vessel wall.^4^ Infected aneurysms are relatively rare and comprise less than 2% of all aortic aneurysms.^3^ Additionally, isolated iliac artery aneurysm/pseudoaneurysms are exceedingly rare with an incidence of 0.03%.^6^

Patient presentations for pseudoaneurysms vary widely as the symptoms depend on size and location of the defect.^4^ They are often painful and can cause local erythema.^5^ Often, presentations are related to secondary effects of the vascular defect and not the defect itself. Infected aneurysms can present with persistent and recurrent fever without clear etiology.^7^ In the modern antibiotic era, the most common organisms are *Salmonella* and *Staphylococcus aureus*; however, a wide range of Gram-positive and Gram-negative organisms has been associated with these infections.^3^ ^,^ ^7^ To date, no case of *Proteus mirabilis* causing an iliac artery pseudoaneurysm associated with direct spread from a prevertebral abscess has been reported.

## CASE REPORT

A 64-year-old-male with a history of type II diabetes mellitus and surgical lumbar decompression in 2013 presented to the emergency department (ED) for left leg swelling. His lumbar decompression was complicated by a methicillin resistant *S aureus* infection requiring multiple surgical washouts and intravenous antibiotics. After surgical washouts in 2013, he was transitioned to suppressive doxycycline until 2019. Shortly after stopping the doxycycline the patient developed a new paraspinal abscess requiring interventional radiology drainage and further antibiotics in 2019. He was placed back on suppressive doxycycline and did well until 2022.

Beginning in late 2022 the patient began to have recurrent fevers and lumbar back pain. He was subsequently diagnosed with a small lumbar paraspinal abscess and was treated with six weeks of vancomycin and gentamicin via a percutaneous indwelling central catheter (PICC). However, his symptoms persisted, and he developed new-onset, isolated left lower extremity edema. This new symptom prompted an ED presentation. This department had no access to the patient’s previous imaging or records except for one computed tomography (CT) of the abdomen and pelvis in the picture archiving and communication system. The patient had reported a possible “groin aneurysm,” but these images were formally reviewed and showed no evidence of aneurysm.

On presentation, the patient reported subjective dyspnea but appeared comfortable in bed. Vital signs showed mild hypertension but no other abnormalities. Exam was remarkable for isolated, non-pitting edema of the left leg from mid-thigh to foot. The left lower extremity was warm, had normal pulses, and no motor or sensory abnormalities.

**Image 1.**
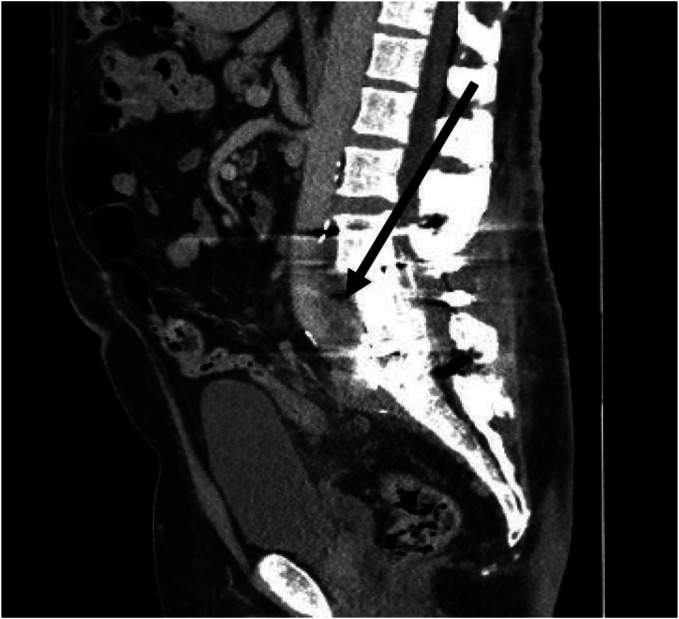
Arrow demonstrating prevertebral abscess spanning the third lumbar to first sacral vertebra. Significant artifact present from spinal hardware.

Initial work-up for deep venous thrombosis (DVT) with Doppler ultrasound was negative. Labs showed no leukocytosis, mild anemia with a hemoglobin 11.5 grams per deciliter (g/dL) (reference range: 13.5–17.0 g/dL) and a C-reactive protein elevated at 68.44 milligrams per liter (mg/L) (0.00–10.90 mg/L). Computed tomography of the chest, abdomen, and pelvis showed moderate left hydronephrosis with mid-ureter obstruction within a prevertebral fluid collection spanning the third lumbar to first sacral vertebra with possible ureter leak. This fluid collection partially encircled the inferior vena cava (IVC) and bilateral common iliac veins and contacted the posterior wall of the infrarenal aorta. It also extended to contact the posterior wall of the rectum where there was concern for possible fistulous connection (Image 1).

Considering these findings, both urology and the orthopedic spine team were consulted. Subsequent magnetic resonance imaging of the spine did not show evidence of an acute process, and no surgical interventions were recommended. Computed tomography cystogram showed no evidence of ureter leak. The urologist felt the obstruction was likely a partial stricture, and the urology team followed the patient during his hospital stay. The patient did not require any urologic intervention.

**Image 2.**
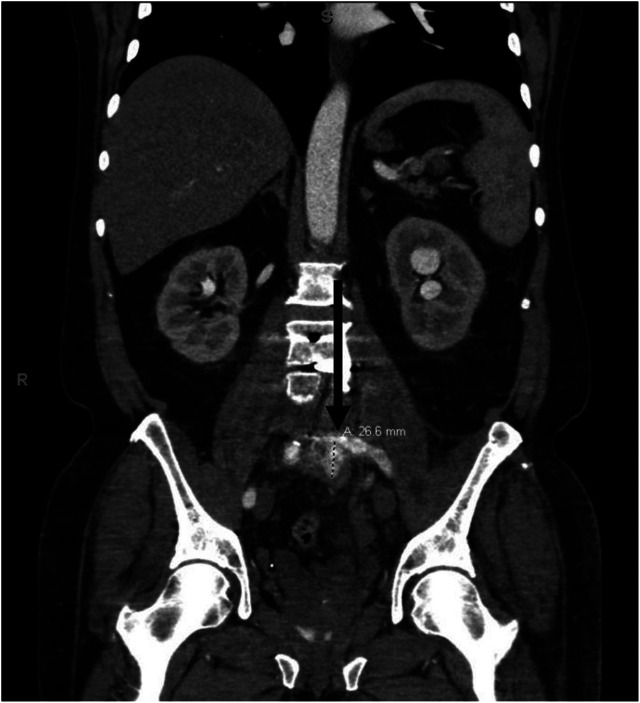
Coronal computed tomography slide with marker indicating 26.6-millimeter left common femoral artery mycotic pseudoaneurysm.

Due to the complexity of the patient and imaging findings, the images were reviewed with the attending radiologist on call who noted possible evidence of left common iliac DVT and possible common iliac arterial wall abnormalities. The decision was made to obtain a dedicated CT angiogram (CTA) of the pelvis. The CTA was concerning for a left common iliac artery mycotic pseudoaneurysm (MPA) within a prevertebral lumbosacral abscess (Image 2).

The patient was taken to the operating room with vascular surgery where he underwent placement of a left common iliac artery stent as well as left femoral artery cutdown and repair. Computed tomography of the abdomen and pelvis with rectal contrast showed no evidence of fistulous connection to the rectosigmoid wall. Definitive repair was completed four days later with right common femoral artery to left superficial femoral artery bypass, IVC filter placement, sartorius myoplasty, and left common iliac artery embolization.

His blood cultures were positive for *P mirabilis,* which was thought to be the culprit of the MPA. His treatment included a prolonged course of vancomycin and ceftriaxone. The patient had a remote history of *S aureus* infection associated with his spinal hardware, but the prevertebral infection was not directly sampled during this presentation.

He was discharged six weeks after presentation with prescriptions for continued home intravenous (IV) antibiotics via PICC. Review of his three-month follow-up appointment revealed the patient was continuing to do well.

## DISCUSSION

Mycotic pseudoaneurysms can occur in multiple different vascular areas and be caused by an array of organisms both bacterial and fungal. They present significant challenges to the clinician and the patients they affect. Diagnosis can be difficult due to their often indolent presentation with non-specific symptoms. Care must be taken when taking a history from patients who may be affected. Identifying possible risk factors for infections (IV drug use, endocarditis, skin and soft tissue infections, vascular grafts, diabetes mellitus) are key to beginning the proper work-up. If they are not diagnosed and treated, they can rapidly lead to significant morbidity and mortality if they rupture.^1^ This case presented a unique patient who was diagnosed with a left common iliac MPA due to *P mirabilis,* which appeared to be a direct complication from an extensive retroperitoneal abscess associated with a previous spinal procedure.

*Proteus mirabilis* is well known for its ability to cause urinary tract infections (UTI) and is often associated with long-term catheterization.^8^ It can cause bacteremia, which is typically associated with an existing UTI. There are reports of *P mirabilis* causing vertebral column infections by hematogenous seeding via the Batson plexus.^8^ This venous plexus consists of valveless veins that allow blood flow between the deep pelvic veins and the internal vertebral veins.^8^ It is much less likely to cause bacteremia from another source, and it is also less likely to be a contaminate in blood cultures, considering it is not skin flora. Literature review did reveal two cases of *P mirabilis*-associated MPA; however, they were both associated with indwelling valves or grafts.^9^ ^,^ ^10^ The patient discussed did have a history of *S aureus* paraspinal infections. Blood cultures were obtained and showed no evidence of other organisms on initial and subsequent testing.

There is sparse data on the occurrence of mycotic aneurysms or pseudoaneurysms. which can be linked to direct bacterial invasion of the vessel wall.^7^ There are some case reports describing spinal-associated infections leading to arterial wall infections in the aorta.^7^ ^,^ ^11^ Literature review showed no known cases of a spinal associated abscess leading to pocket expansion and subsequent involvement of a common iliac artery.

Another interesting feature of this case is the relation of the presenting complaint (unilateral leg swelling) with the ultimate diagnosis (mycotic aneurysm due to *P mirabilis)*. Sensitivities of venous ultrasound for DVT have been reported between 62–94%.^12^ ^,^ ^13^ This wide variation depends on the type of ultrasound performed (compression, duplex, and triplex) and the site(s) involved. Indirect CT venography has been found to better visualize proximal DVTs in the large pelvic veins.^14^ For patients in whom there is a high suspicion for a proximal DVT and negative DVT ultrasound, indirect CT venography would be a reasonable next step. Determining the best imaging for complicated patients can be difficult. A discussion with a radiologist about the specific concerns surrounding a patient can help select the best modality of CT and assist in accurate contrast timing to delineate the high-yield structures.

Mycotic aneurysms may exert mass effect on or fistulize with surrounding structures, including adjacent deep veins, ureters, and bowel.^15^ This patient’s initial presentation was due to unilateral leg swelling from the DVT associated with the MPA. The symptoms most associated with mycotic aneurysm are nonspecific and include fever, pain over the affected vascular site and, rarely, septic shock.^16^ This case highlights the importance of maintaining a high index of suspicion in patients who present with unexplained symptoms of mass effect (hydroureter, unilateral leg swelling) and occult infection (historical risk and subacute systemic infectious symptoms), even when initial and routine imaging does not demonstrate causative pathology.

This case involves multiple different imaging modalities and demonstrates the importance of selecting the correct modality for the suspected abnormality. This patient had multiple CT images prior to the angiogram, which was ultimately diagnostic. Discussions with the attending radiologist reviewing the imaging revealed the detail needed to see the MPA was only obtained through the contrast timing associated with a CT angiogram. The routine contrasted scan did not allow for the detail needed to diagnose the patient’s MPA.

## CONCLUSION

The patient discussed in this case report had a unique and rare presentation of an already rare disease process. Mycotic pseudoaneurysms alone are rare, but this case was associated with an uncommon organism and presentation that was further complicated by lack of access to historical surgical records. While making the diagnosis of a MPA is difficult, missing it can lead to devastating outcomes for patients. This patient’s history included important risk factors and symptoms, which indicated the possible underlying etiology. A high clinical suspicion is needed to accurately diagnose these cases. To accurately diagnose this life-threatening condition our patient received multiple imaging modalities and consultations during an extended ED stay. Treating clinicians in the ED must realize that the uncovering of a MPA is a tough diagnostic challenge that is not made quickly. Clinical perseverance will be needed to make an accurate and critical diagnosis.
